# RNA-Seq-Based Analysis of the Physiologic Cold Shock-Induced Changes in *Moraxella catarrhalis* Gene Expression

**DOI:** 10.1371/journal.pone.0068298

**Published:** 2013-07-02

**Authors:** Violeta Spaniol, Stefan Wyder, Christoph Aebi

**Affiliations:** 1 Institute for Infectious Diseases, University of Bern, Bern, Switzerland; 2 Department of Clinical Research, University of Bern, Bern, Switzerland; 3 Department of Pediatrics, University of Bern, Inselspital, Bern, Switzerland; Cornell University, United States of America

## Abstract

**Background:**

*Moraxella catarrhalis*, a major nasopharyngeal pathogen of the human respiratory tract, is exposed to rapid downshifts of environmental temperature when humans breathe cold air. The prevalence of pharyngeal colonization and respiratory tract infections caused by *M. catarrhalis* is greatest in winter. We investigated how *M. catarrhalis* uses the physiologic exposure to cold air to regulate pivotal survival systems that may contribute to *M. catarrhalis* virulence.

**Results:**

In this study we used the RNA-seq techniques to quantitatively catalogue the transcriptome of *M. catarrhalis* exposed to a 26°C cold shock or to continuous growth at 37°C. Validation of RNA-seq data using quantitative RT-PCR analysis demonstrated the RNA-seq results to be highly reliable. We observed that a 26°C cold shock induces the expression of genes that in other bacteria have been related to virulence a strong induction was observed for genes involved in high affinity phosphate transport and iron acquisition, indicating that *M. catarrhalis* makes a better use of both phosphate and iron resources after exposure to cold shock. We detected the induction of genes involved in nitrogen metabolism, as well as several outer membrane proteins, including *ompA*, *m35*-like porin and multidrug efflux pump (*acrAB*) indicating that *M. catarrhalis* remodels its membrane components in response to downshift of temperature. Furthermore, we demonstrate that a 26°C cold shock enhances the induction of genes encoding the type IV pili that are essential for natural transformation, and increases the genetic competence of *M. catarrhalis*, which may facilitate the rapid spread and acquisition of novel virulence-associated genes.

**Conclusion:**

Cold shock at a physiologically relevant temperature of 26°C induces in *M. catarrhalis* a complex of adaptive mechanisms that could convey novel pathogenic functions and may contribute to enhanced colonization and virulence.

## Introduction

*Moraxella catarrhalis* colonizes the mucosal surface of the human nasopharynx and is a major cause of acute otitis media in children and of exacerbations of chronic obstructive pulmonary disease in adults [1], [2]. The routine use of pneumococcal conjugate vaccines has altered nasopharyngeal colonization patterns and caused an increased prevalence of colonization and infection by *M. catarrhalis* [3], [4]. Moreover, clinical studies revealed that the prevalence of pharyngeal colonization and respiratory tract infections caused by *M. catarrhalis* displays seasonal variation and increases in winter [5], [6], [7], [8]. Viral infections occurring during the cold season pave the way for subsequent bacterial secondary infection by T-cell mediated release of interferon-γ, which inhibits bacterial phagocytosis by macrophages [9], and increases the expression of adhesion receptors on epithelial cells such as carcinoembryonic antigen-related cell adhesion molecule 1 (CEACAM1) [10]. In addition, the human nasopharyngeal flora is repeatedly exposed to rapid downshifts of environmental temperature. Breathing cold air (e.g., −1°C at 10–20 l/min) reduces the nasopharyngeal temperature from 34°C at room temperature to about 26°C within several minutes and for extended periods of time [11]. Such rapid downshift of temperature induces adaptive events in the residential upper respiratory tract flora that may contribute to the transition from asymptomatic colonization to bacterial secondary infection.

Our previous studies demonstrated that a 26°C cold shock up-regulates the expression of important virulence traits, such as adherence to epithelial cells, iron acquisition, complement resistance and immune evasion [12], [13]. An increased expression of the UspA1 adhesin on the surface of *M. catarrhalis* at 26°C leads to an increased adherence to upper respiratory tract epithelial cells *in vitro* [12], [14]. Exposure of *M. catarrhalis* to 26°C also increases the outer membrane protein (OMP)-mediated release of the proinflammatory cytokine IL-8 in pharyngeal epithelial cells [12] and enhances the expression of genes involved in the acquisition of transferrin and lactoferrin [13]. In contrast, cold shock decreases the expression of hemagglutinin (*hag/mid*), a major lower respiratory tract adhesin, which mediates B cell response, and reduces immunoglobulin D-binding on the surface of *M. catarrhalis*. Furthermore, cold shock reduces the expression of porin M35, which may affect the resistance to aminopenicillins [15]. Thus, the physiologic cold shock appears to be an important contributor to the seasonal peak in *M. catarrhalis* infections and could be clinically relevant during the cold season by temporarily increasing the organism’s virulence.

In the present study, we performed a comprehensive transcriptome analysis in *M. catarrhalis* exposed to 37°C or to a 26°C cold shock using the Illumina® RNA-seq technique. We were able to define global changes in gene expression in response to cold shock. We observed that a 26°C cold shock induces the expression of genes that in other bacteria have been related to virulence. These data provided broader and deeper insights into the transcriptional regulation in *M. catarrhalis* after a 26°C cold shock and also provide a rich basis for further investigations.

## Materials and Methods

### Bacterial Strains and Culture Conditions

*M*. *catarrhalis* strain O35E and the clinical isolate 415 have been described elsewhere [13], [14]. Bacteria were cultured at 37°C and 200 rpm in brain heart infusion (BHI) broth (Difco) or on BHI agar plates in an atmosphere containing 5% CO_2_. Cold shock experiments were performed as described [14]. Bacteria were grown overnight at 37°C, resuspended in fresh medium and grown to mid-logarithmic phase (optical density at 600 nm [OD_600_] of 0.3). Subsequently, bacteria were exposed to 26°C or 37°C, respectively, for 3 hours.

### RNA Preparation and Illumina RNA-seq

*M*. *catarrhalis* cultures were fixed with 2 volumes of RNA protect (Qiagen) and harvested. RNA was isolated using the RNeasy kit (Qiagen) as described elsewhere [14]. Residual DNA in the samples was removed using Dnase I. The integrity of the RNA was analyzed using an Agilent bioanalyzer (Agilent technologies). The Ribo-Zero rRNA removal kit (Gram-negative bacteria, Epicentre) was used to remove the 23S and 16S rRNA from the total RNA samples. Library construction and sequencing were performed by the Genomics Core Facility of the Department of Clinical Research, University of Bern using TruSeq RNA sample preparation v2 guide (Illumina). Sequencing raw data generated in this study are available at GEO, accession number GSE46256 (http://www.ncbi.nlm.nih.gov/geo/query/acc.cgi?acc=GSE46256).

### RNA-seq Data Analysis

The quality analysis of RNA-seq data was carried out using the FastQC application (http://www.bioinformatics.babraham.ac.uk/projects/fastqc/). The *M*. *catarrhalis* O35E genome sequence as well as open reading frame (ORF) positions and annotations were obtained using the RefSeq and PATRIC database [16] (http://patricbrc.vbi.vt.edu/portal/portal/patric/Home and http://brcdownloads.vbi.vt.edu/patric2/genomes/Moraxella_catarrhalis_O35E). Reads were mapped to the *M*. *catarrhalis* O35E RefSeq genome sequence using the BWA algorithm [17]. The summarization of counts was performed using the BEDTools software [18], and normalization and the differential expression analysis was performed using the variance analysis package DEseq [19] using R version 2.8.0. The total read count was determined for each gene by combining data from three biological replicate sequencing runs. *P*-values were calculated and adjusted for multiple testing using the false discovery rate (FDR) controlling procedure [20]. Functional classification of the *M*. *catarrhalis* O35E genes was projected from the *M*. *catarrhalis* RH4 genome [21] by identifying the orthologous proteins between these strains using the best reciprocal blast hit approach with protein sequences. For all but seven proteins from the O35E strain an 1∶1 orthologous protein could be identified in the RH4 strain. The seven O35E proteins were equally similar to multiple (2 or 3) different RH4 proteins. Enrichment analysis was performed using the functional categories with a custom python skript (Fisher’s exact test) correcting for multiple testing using the FDR.http://www.nature.com/nature/journal/v486/n7402/full/nature11234.html - ref12.

### Quantitative Reverse Transcriptase PCR (qRT-PCR) Assays

RNA was isolated as described in the previous section. As templates for this assay we used the one set of RNA samples that was used for the synthesis of first Illumina library and two additional pairs of samples that were independently obtained. The reverse transcription step was carried out using the Superscript II cDNA synthesis kit (Invitrogen) according to the manufacturer’s instructions. Oligonucleotide primer pairs (Table S1) were designed for use in qRT-PCR with either PrimerExpress software (Applied Biosystems) or Primer 3 [22]. To assess for DNA contamination, RNA samples were also run without reverse transcriptase. The constitutively expressed 16S ribosomal RNA gene was used as an internal control for relative quantification. Reactions for qRT-PCR were completed using *Power* SYBR green PCR Master Mix (Applied Biosystems) with a two-step reaction protocol consisting of 40 cycles of 94°C for 30 s and 60°C for 1 min, followed by a dissociation phase for quality control. The 25-µl qPCR mixtures contained 0.2 µM specific primers, Power SYBR green (Applied Biosystems) (2x), and 2 µl of cDNA (10 ng/µl). Relative quantification of gene expression was performed using the comparative threshold method. The ratios obtained after normalization were expressed as folds of change compared with samples isolated from bacteria exposed to 37°C.

### Natural Transformation

The quantitative transformation assay was performed as described elsewhere [23]. Briefly, *M. catarrhalis* cells exposed to 37°C or to a 26°C were adjusted to an OD_600nm_ of 0.2, glycerol was added to a final concentration of 20% and 200 µl aliquots of these naturally competent cells were stored at −70°C until use. Aliquots (100 µl) were spread on BHI agar plates and air-dried. Thirty µl of solution containing 100 ng of purified linear DNA (*M. catarrhalis uspA1*::*kan*) was spotted onto the lawn in duplicate for each condition, followed by incubation at 26°C or 37°C, 5% CO_2_. After 5 h, bacteria were harvested and resuspended in 1 ml of BHI broth. Serial dilutions were prepared and spread on BHI agar plates for total viable cell counts, or on BHI agar plates containing kanamycin at 20 mg/l. After 24 h of incubation at 37°C in 5% CO_2_, the number of CFUs per experiment was determined and transformation frequencies were calculated. All experiments were performed in independent triplicates.

### Antimicrobial Peptide Susceptibility Assay

Antimicrobial peptide susceptibility assay was performed as described elsewhere [24]. Bacteria were exposed either to 26°C or 37°C for 3 h, adjusted to approximately 5×10^5^ CFU/ml in 100 mM NaCl, 2% BHI and 10 mM PBS (pH 7). Aliquots (5 µl) of this suspension were mixed in 1.5-ml microcentrifuge tubes with various concentrations of polymyxin B (Sigma). In all cases the final volume was 30 µl. After 1 h of incubation at the bacterial growth temperature, the contents of the tubes were plated onto BHI agar. Colony counts were determined, and results were expressed as percentages of the colony count of bacteria not exposed to the antibacterial agent.

### Statistical Analysis

Data were expressed as mean ±1 standard deviation (SD). Differences between groups were analyzed by one-way analysis of variance with a Bonferroni correction using Prism software (version 5.01; GraphPad). *P*<0.05 was defined as statistically significant.

## Results and Discussion

### Sequencing of the *M. catarrhalis* Transcriptome after Exposure to 26°C and 37°C

**Figure 1:**
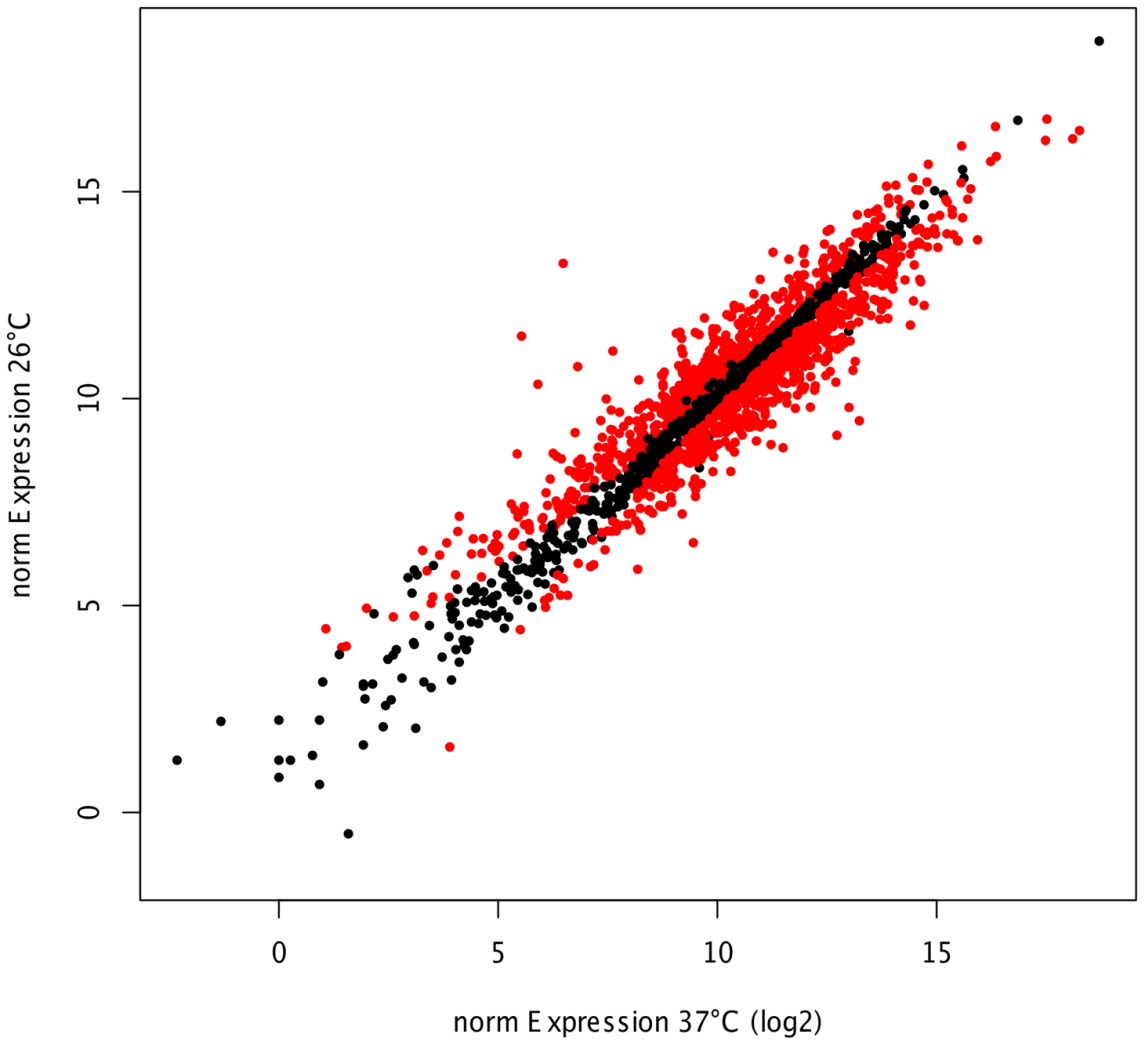
Differentially expressed genes following cold shock. The scatterplot shows normalized counts for *M. catarrhalis* either grown at 26°C or 37°C (log2 scale, mean of three biological replicates). Each dot represents an individual gene. Genes showing significant differential expression in the RNA-seq data are highlighted in red, genes that are not regulated by temperature are highlighted in black. The genes are highlighted according their adjusted p-value (False Discovery Rate).

**Figure 2:**
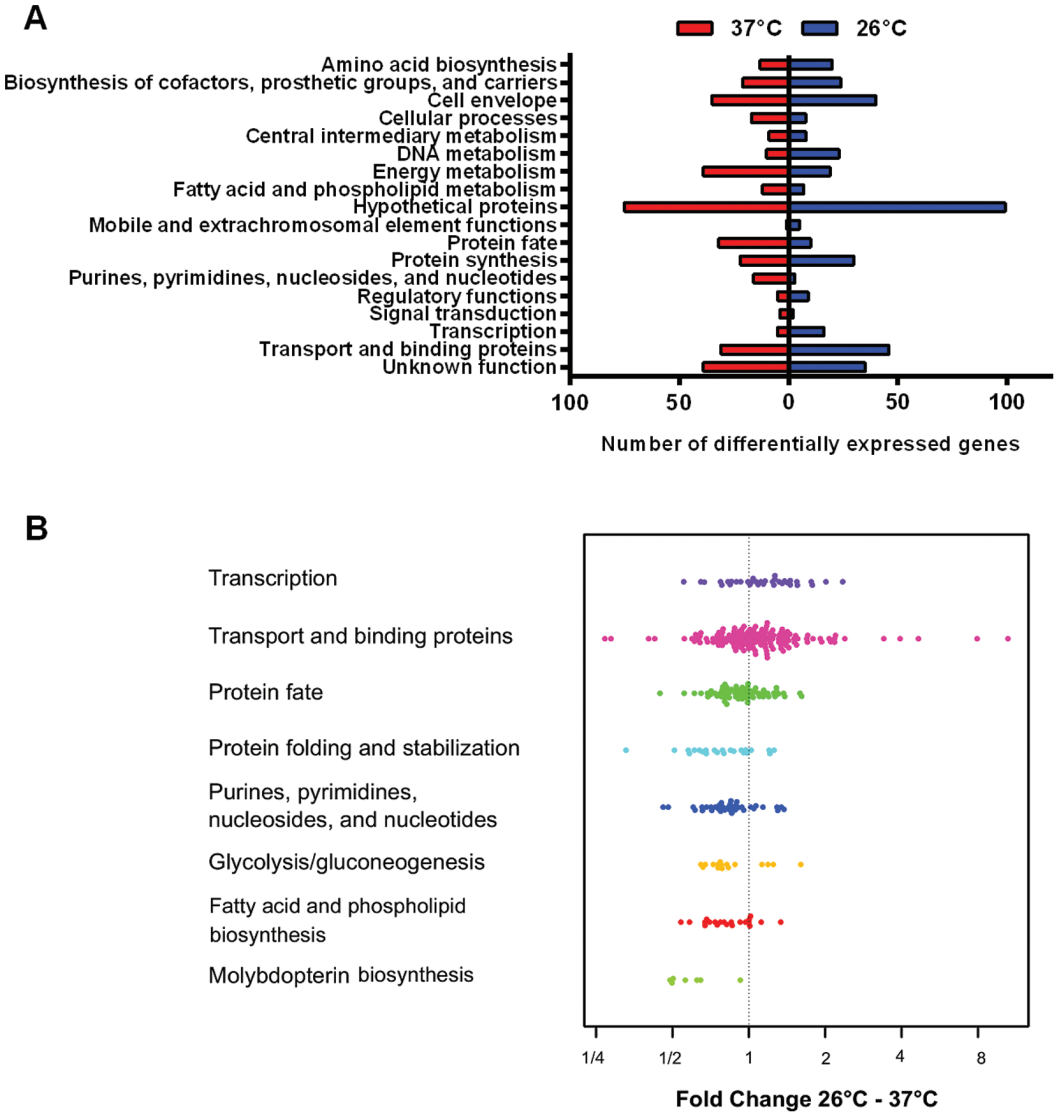
Functional categories of *M.catarrhalis* genes induced or repressed following cold shock. A. Differentially expressed genes (Table S4) were broadly categorized according to their biological function. Each bar represents the actual number of genes. Blue bars indicate number of genes that are upregulated at 26°C, and red bars represent number of genes that are downregulated at 26°C. B. The sets of up- or downregulated genes (based on DESeq analysis) were tested for enrichment in specific functional categories. Average log2 fold-changes of individual genes are shown for categories with adjusted p-values<5%.

Whole genome mRNA sequencing was used to monitor the global changes in gene expression of *M. catarrhalis* strain O35E exposed to a physiological 26°C cold shock relative to continuous growth at 37°C. In order to determine the cold shock stimulon, RNA-seq was performed on three biological replicates of *M. catarrhalis* growth at different temperatures using the Illumina HiSeq 2000 system. Since 16S and 23S rRNA were anticipated to be the most abundant RNA species, these were depleted prior to sequencing by oligonucleotide hybridisation-mediated capture. A total of 183 million single-end reads (100 bp) was obtained of which 47′900′062 reads mapped uniquely to the *M. catarrhalis* O35E strain genome. Between 3.2 and 14.5 million reads for each sample aligned to non-rRNA regions of the *M. catarrhalis* genome representing approximately 25% of all reads (Table S2), which illustrates the suitability of RNA-seq for bacterial transcriptomic studies. When expression data for each biological replicate were plotted against each other, the normalized counts were highly reproducible (Figure S1). RNA expression levels of *M. catarrhalis* replicates upon exposure to 26°C and 37°C were highly similar (R^2^>0.97 and R^2^>0.99, respectively). Genes with differential expression between 26°C and 37°C were identified using the DEseq [19] package (Table S3). Using a FDR cut-off of 5% a total of 831 genes were significantly differentially expressed greater than 1.5-fold, including 429 up-regulated genes and 402 down-regulated genes at 26°C (Table S4). Differential expression was defined according to *P* value for each gene by the comparison of normalized counts (log2 scale, average of 3 replicates) between 26°C and 37°C (Figure 1). The magnitude distribution of the significantly differently expressed genes was illustrated by MA plot analysis showing individual gene responses plotted as log2 fold-change versus mean expression (Figure S2). Next we analyzed whether cold shock-regulated gene transcripts were enriched in specific biological processes (Figure 2A). Among the differentially expressed genes we identified several statistically enriched functional categories (Figure 2B and Table S5). The following categories were enriched among gene transcripts induced upon a cold-shock of 26°C: “Transcription” and “Transport and binding proteins”. Functional categories enriched among genes repressed at 26°C included: “Protein fate”, “Protein folding and stabilization”, “Purines, pyrimidines, nucleosides, and nucleotides”, “Glycolysis/gluconeogenesis”, “Fatty acid and phospholipids biosynthesis” and “Molybdopterin biosynthesis”.

### Validation of Differentially Regulated Genes by qRT-PCR

**Figure 3:**
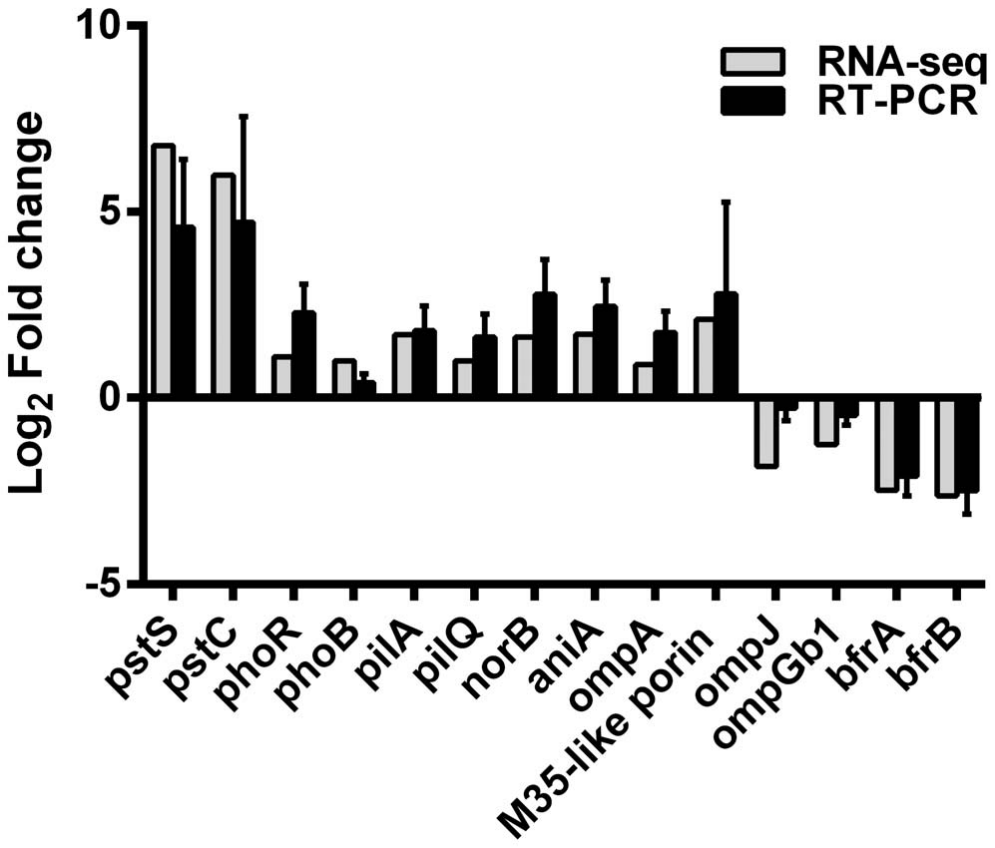
qRT-PCR validation of differentially expressed genes. Fold changes of the expression levels of genes *pstS* encoding phosphate ABC transporter, periplasmic phosphate-binding protein PstS; *pstC* encoding phosphate transport system permease protein PstC; *phoR* encoding two-component system phosphate regulon sensor histidine kinase PhoR; *phoB* encoding two-component system phosphate regulon response regulator PhoB; *pilA* encoding type IV pilin PilA; *pilQ* encoding type IV pilus secretin PilQ; *norB* encoding nitric oxide reductase NorB; *aniA* encoding nitrite reductase AniA/Msp78; *ompA* encoding OmpA family protein; *m35-like porin* encoding M35-like porin protein, *ompJ* encoding outer membrane protein J, *ompG1b* encoding outer membrane protein G1b; *bfrA* and *bfrB* encoding bacterioferritins were compared to those detected by RNA-seq. The log2 of the fold difference in gene expression between 26°C and 37°C temperatures as determined by qRT-PCR (black columns) is plotted adjacent to the results obtained in RNA-seq analysis (grey columns). The results of qRT-PCR are expressed as means ±1 SD of three separate experiments performed in duplicate.

Several genes found to be differentially expressed by RNA-seq have been already identified in our previous studies [13], [14], [15]. For example, a 26°C cold shock induced expression of genes encoding a major adhesin UspA1 (*uspA1*), genes involved in transferrin and lactoferrin acquisition (*tbpA/B*, *lbpA/B*) and reduced the expression of genes encoding a M35-porin (*m35*) and hemagglutinin (*hag/mid*). To further corroborate the RNA-seq data, several differentially expressed genes were selected for secondary confirmation using quantitative RT-PCR analysis. As shown in Figure 3, qRT-PCR experiments confirmed that the expression of these genes was regulated by temperature. The fold changes detected for each gene were similar to the fold changes observed by RNA-seq. A similar expression profile was observed when *M. catarrhalis* clinical isolate 415 was exposed to 26°C and 37°C, with the exception that no transcription of the gene encoding the m35-like protein was found (data not shown).

### Differentially Expressed Genes Involved in Transcription and Replication

Cold causes formation and stabilization of secondary structures in RNA, which will interfere with efficient ribosomal binding, elongation and translation termination [25]. Bacteria have evolved several established strategies to counteract the deleterious influence of cold-induced mRNA structuring. One of them is based on the cooperative binding of RNA chaperones (CspA family) to nascent mRNA transcripts for preventing the formation of mRNA secondary structures. This process leads to preferential translation of cold-inducible mRNA [25]. The CspA family in *E. coli* comprises several homologous proteins that are cold inducible [26]. Two cold shock proteins, CspA and cold shock-like protein, were predicted by genome analysis of *M. catarrhalis* [21]. Our data demonstrate that cold shock strongly enhanced (4.8-fold) the transcription of cold shock-like protein, whereas the expression of the gene encoding cold shock protein CspA was only moderately (1.4-fold) increased.http://www.sciencedirect.com/science/article/pii/S1286457905000079 - bib34#bib34 The ATP-dependent RNA helicase-encoding genes (*rhlB*), whose transcription was enhanced (1.9-fold) after temperature downshift, are known to be involved in the resolving of the cold-induced structuring of mRNA [27].

Cold shock causes a severe impairment of the translation machinery, leading to a block in translation initiation and decoupling of transcription to translation [28]. Several genes (*infB*, *rho, nusAB*) involved in modulating of translation have been identified to be cold shock inducible, indicating that activation of translation is accomplished by enhanced expression of translation factors and components specifically associated with the ribosome. We found that numerous genes involved in the transcription process were significantly induced after exposure to cold shock (Figure 2B and Table S5) including several transcription factors (*dksA, rho, nusA/B, nadR*) and DNA-dependent RNA polymerases (*rpoA, rpoC*).

Low temperature affects the conformation, flexibility and topology of DNA, which in turn have a deleterious influence on DNA replication [29]. In our study, several genes involved in DNA replication, recombination, and repair (*dnaA/E*, *recN/G, topA*) as well as genes involved in restriction/modification were identified to be up-regulated upon cold shock.

Among the down-regulated genes upon shift from 37°C to 26°C were genes encoding major heat shock proteins GroES, GroEL and DnaK. This suggests that under conditions of a 26°C cold shock, bacteria no longer require elevated expression of these genes.

The analysis of potential transcriptional regulators within the *M. catarrhalis* genome revealed, that cold shock increased the expression of several regulatory DNA-binding proteins including cold shock-like protein (4.8-fold), nitrite-sensitive transcriptional repressor NsrR (4.8-fold), LysR (2.2-fold) and the AsnC (2.2-fold) family transcriptional regulators. The two-component system phosphate regulon response regulator PhoB (2-fold) and sensor histidine kinase PhoR (2.1-fold) were also induced following cold shock. However, two TetR family transcriptional regulators (1.6 and 1.8-fold), two-component system nitrate/nitrite response regulator NarL and sensor histidine kinase NarX were upregulated (2.3- and 1.7-fold, respectively) at 37°C.

### Differentially Expressed Genes Involved in Carbon und Energy Metabolism

Generally, genes involved in energy metabolism particularly in glycolysis/gluconeogenesis and electron transport were expressed at a greater level after exposure of *M.catarrhalis* to 37°C (Figure 2A and 2B, Table S5). *M. catarrhalis* possesses an incomplete glycolytic pathway, indicating the inability to utilize exogenous carbohydrates [30]. All of the enzymes of the gluconeogenic pathway are present, suggesting that carbohydrate intermediates can be synthesized [21], [31]. Gluconeogenesis uses phosphoenolpyruvate (PEP) as a starting substrate, which can be generated from tricarboxylic acid (TCA) cycle intermediates. The expression of numerous glycolytic enzymes (*fbp, gapA, gpmI, eno, ppsA, aceE*), D-lactate dehydrogenase (*dld*) and L-lactate dehydrogenase (*lldD*) was at a greater level at 37°C. Acetate kinase (*ackA*), required for conversion of acetate into acetyl-CoA, was also upregulated at 37°C. In contrast, triose-phosphate isomerase (*tpiA*) that catalyses the reversible conversion of glyceraldehyde-3-phosphate and dihydroxyacetone and glucose-6-phosphate isomerase (*pgi*) were upregulated after exposure to 26°C.

The TCA cycle is supplied with acetyl coenzyme A (acetyl-CoA) via degradation of fatty acids and acetate assimilation. The enzymes of the TCA cycle were induced after exposure to cold shock conditions. Genes encoding succinate dehydrogenase (*sdhA*), aconitate hydratase (*acnB*) and enzyme of the glyoxylate bypass (*aceB*), that are required for utilization of acetyl-coenzyme A (CoA) during gluconeogenesis, were upregulated after temperature shift to 26°C. The malic enzyme (*maeB*), which converts malate to the level of pyruvate, which can enter the TCA cycle, was upregulated after exposure to 37°C. Studies on *Yersinia pestis* showed that a shift from 26°C to 37°C caused downregulation of numerous glycolytic enzymes (*gapA*, *gmpA*, *eno*), whereas the carbohydrate transport systems (eg. ABC galactose transporter, *mglBAC*) and a full TCA cycle were upregulated at 37°C [32].

The majority of genes encoding enzymes involved in aerobic respiration and electron transport was enhanced after continuous exposure of *M. catarrhalis* to 37°C, including the cytochrome c oxidase subunits (*ccoGPS*), cytochrome c-type biogenesis protein (*ccmE*), cytochrome d ubiquinol oxidase subunits (*cydAB*), thioredoxin (*trx*) and thioredoxin reductase (*trxR*).

Several genes encoding enzymes involved in aerobic respiration and electron transport were also induced upon cold shock, including the rubredoxin family protein, NADH dehydrogenase subunits (*nuoEG*) and 4Fe-4S/2Fe-2S ferredoxins. Exposure *M. catarrhalis* to a 26°C cold shock thus can induce the energy generation, which ensures ATP-dependent responses such as removing RNA secondary structures with ATP-dependent RNA helicases and increased degradation of fatty acids during cold adaptation.

### Differentially Expressed Genes Involved in Lipid Metabolism

Shift to 26°C caused in *M. catarrhalis* the induction of several genes encoding enzymes (*fadE, fadJ, fadD/fadK, pgpA*) involved in fatty acid degradation that subsequently supplies the TCA cycle with acetyl coenzyme A (acetyl-CoA). However, cold shock reduced the expression of several genes (*fabB*, *fabH, accABCD*) encoding enzymes or regulators involved in fatty acid biosynthesis. Furthermore, outer membrane protein E (*ompE*), a putative FadL homolog and long-chain-fatty-acid transporter is induced (2.3-fold) after exposure to 37°C. The transcription of *Y. pestis* genes (*fabF*/*fabJ*, *fabH*, *fabG*, *fabE*, and *fadR*), encoding enzymes or regulators involved in fatty acid biosynthesis, were induced by a 10°C cold shock, indicating the increase of fatty acid biosynthesis during *Y.* *pestis* cold adaptation [33].

### Differentially Expressed Genes Involved in cell Envelope Biosynthesis

Lipooligosaccharide (LOS) is a major component of the outer leaflet of the outer membrane of gram-negative bacteria. In this study, the mRNA level of several genes (*lpxB*, *lpxX*, *kdtA* and *lgt6*) involved in LOS biosynthesis was enhanced by cold shock. Similarly, *Y. pestis* comprises several homologous proteins (*lpxA, lpxB, kdtA*) that are cold inducible [32], [33]. In contrast, we found that the expression of genes encoding UDP-3-O-N-acetylglucosamine deacetylase (*lpxC*) and dodecanoyltransferase (*lpxL*) were enhanced after exposure to 37°C. LpxB is a key enzyme participating in lipid A biosynthesis. The Kdo transferase gene *kdtA* catalyzes the addition of Kdo residues to the lipid A precursor. The *lpxL* and *lpxX* genes encode late acyltransferases that are responsible for the incorporation of secondary acyl chains into lipid A. The expression levels of the acyltransferases are temperature regulated, and the lipid A acylation status affects the expression of *Yersinia enterocolitica* virulence factors [34]. Decreased acylation allows the bacteria to evade detection by the host innate immune system. Induction of the innate immune system by modifying the tetraacylated lipid A structure by overexpression of the late acyltransferase LpxL in *Y. pestis* results in a complete loss of virulence [35]. Previous studies demonstrated that *Y. enterocolitica* is more susceptible to polymyxin B, a model of antimicrobial peptides of the innate immune system, when grown at 37°C than at 22°C by interacting with the anionic lipid A moiety of the LPS [24]. Therefore, we hypothesized that temperature may also regulate the expression of *M. catarrhalis* lipid A modifications linked to antimicrobial peptides resistance. However, we found no differences in the susceptibility of *M. catarrhali*s O35E exposed to a 26°C cold shock or to continuous growth at 37°C to polymyxin B (data not shown), indicating that a 26°C may not be sufficiently low to induce remarkable changes in the LOS structure, or that the reciprocal action of the *M. catarrhali*s LpxX and LpxL acyltransferases may compensate this effect.

The peptidoglycan layer is the main target for ß-lactam antibiotics that can be degraded by the Bro beta-lactamases produced by *M. catarrhalis* [36]. The transcription of gene encoding the beta-lactamase family protein (*bro-1*) involved in resistance was increased (2.5-fold) at 37°C.

Type IV pili (TFP) have a wide variety of functions, including adhesion to epithelial cells, biofilm formation and motility, and play a crucial role in the initiation of disease by a wide range of human mucosal pathogens [37]. Furthermore, expression of TFP by *M. catarrhalis* is essential for natural competence [38]. This mechanism of DNA transformation is a major contributor to the horizontal exchange of genetic information between naturally competent microorganisms [39]. Analysis of RNA-seq data demonstrated that a 26°C cold shock caused in *M. catarrhalis* the induction of genes encoding type IV pilin A (*pilA,* 3.2-fold), type IV pilus secretin (*pilQ*, 2.0-fold) and several type IV pilus biogenesis proteins (*pilBCDT*). Moreover, the transcription of genes encoding major competence proteins (*comEA, comM*) and the DNA protecting protein (*dprA*) was strongly induced in *M. catarrhalis* by cold shock. It has been demonstrated, that the type IV A pilin-encoding gene, *pilA*, expression of *Burkholderia pseudomallei* was dramatically increased following growth at 27°C compared to growth at 37°C [40]. In contrast, the surface expression of the *Enterococcus faecium* PilA- and PilB-type pili was regulated in a temperature-dependent manner, as polymerization of two distinct types of pili at the surface only occurred when cells were grown at 37°C; no pili were observed on cells grown at 21°C [41]. Similarly, expression of *pil* operon was strongly induced after growth of *Y. pseudotuberculosis* at 37°C as compared to growth at 28°C [42].

Increased methylation state has been shown to enhance the transformation efficiency in *Neisseria gonorrhoeae* protecting foreign DNA from digestion by restriction enzymes [43]. The expression of gene encoding the type III restriction system methylase was strongly induced at 26°C (5.8-fold) in comparison to 37°C suggesting that cold shock increases the transformation efficiency also by protecting foreign DNA from own restriction modification systems.

**Figure 4:**
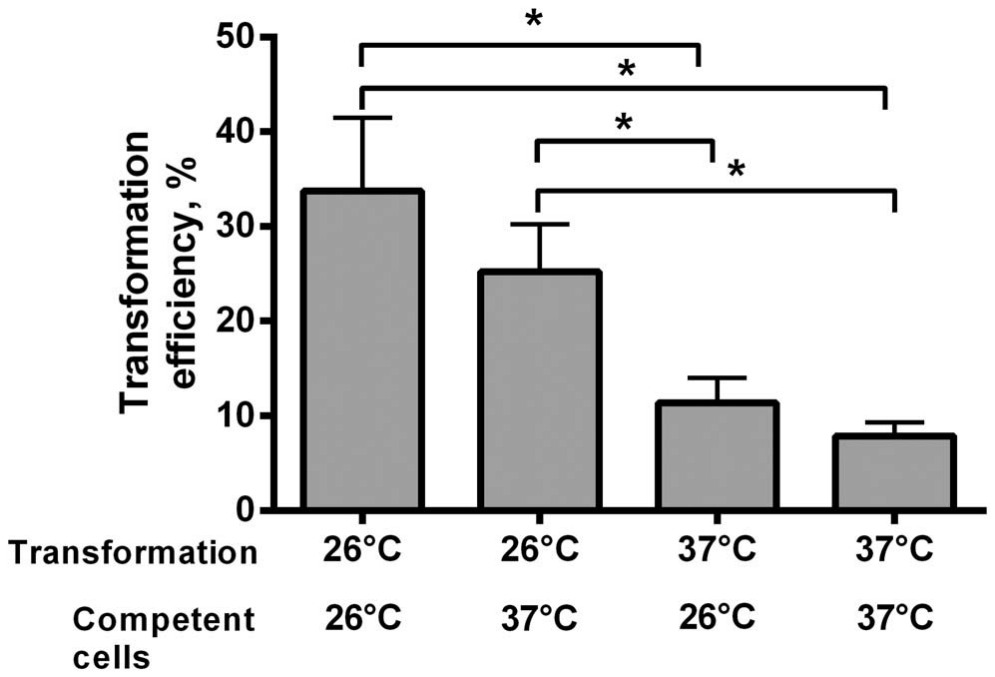
Influence of temperature on natural transformation of *M. catarrhalis*. *M. catarrhalis* strain O35E, growth at 26°C or 37°C, was transformed with 100 ng of purified linear DNA (*M. catarrhalis uspA1*::*kan*) and incubated at 26°C or 37°C for 5h. Transformation frequencies are expressed as the transformant CFU per total CFU and represent the average of three independent assays performed in duplicates. *, *P*<0.05, one-way analysis of variance.

Because the transcription of type IV pili was found to be induced after incubation at 26°C, and the expression of TFP is correlated with natural genetic transformation in many bacterial species, we investigated whether cold shock conditions affect the natural genetic competence of *M. catarrhalis*. The contribution of cold shock to *M. catarrhalis* DNA competence was investigated in a quantitative transformation assay. *M. catarrhalis* strain O35E was exposed to 26°C or 37°C and evaluated for their differences in the natural transformation frequencies. The DNA transformation experiments revealed that *M. catarrhalis* following a 26°C cold shock show greater (more than 4-fold) natural transformation frequencies than bacteria exposed to 37°C (Figure 4). The natural genetic competence of *M.catarrhalis* was also increased (3-fold) after cold shock in bacteria during planctonic growth (data not shown). Similarly, cold shock increases (2.1-fold) the competence of *M.* *catarrhalis* clinical isolate 415 (data not shown).

Analysis of RNA-seq data demonstrated that a 26°C cold shock caused in *M. catarrhalis* a significant induction of numerous genes encoding transport and binding proteins (Figure 2B and Table S5). Examples are OmpA family protein (1.8-fold), M35-like porin (4.3-fold) as well as systems involved in transport of amino acids, peptides and amines like oligopeptide ABC transport system (*oppBDF*), arginine ABC transporter (*artQ*), amino acid ABC transporter (*occM*), systems involved in anions transport like phosphate ABC transporter system (*pstACS*), nitrate ABC transporter (*nrtABCD*), systems involved in transport of cations and iron carrying compounds like transferrin and lactoferrin binding proteins (*tbpAB, lbpAB*), acetate permease ActP (cation/acetate symporter), chelated iron ABC transporter (*afeBCD*) and motA/TolQ/ExbB proton channel. It has been shown that in *E.coli* human body temperature increases amino acid, carbohydrate and iron uptake and utilization gene expression compared to growth at 23°C [44]. A major outer membrane protein A (OmpA) of *Acinetobacter baumannii* plays a partial role in biofilm formation and is absolutely required for attachment to epithelial cells [45]. Several lines of evidence have shown that the OmpA of *E. coli* and *Klebsiella pneumoniae* can protect bacteria from bactericidal effects [46], [47].

### Differentially Expressed Genes Involved in Protein Fate

Many genes encoding proteins associated with protein fate were found to be induced after exposure to 37°C (Figure 2B and Table S5). Especially the genes encoding proteins involved in protein folding and stabilization displayed increased expression at 37°C. Two protein secretion systems (Tat and Sec) that transport proteins from the cytosol across the inner membrane to the periplasm were found in the *M. catarrhalis* genome [21]. Genes encoding the Tat translocation proteins (*tatAB*) were induced at 37°C. In contrast, a 26°C cold shock caused in *M. catarrhalis* the induction of genes encoding the Sec protein secretion system (*secGY*) as well as genes encoding components of the resistance-nodulation-division (RND) multidrug efflux systems (*acrAB*, *oprM*) [48].

### Differentially Expressed Genes Involved in Nitrogen Metabolism

The denitrification pathway of *M. catarrhalis* provides an alternative mechanism to generate energy under lower oxygen tension and contributes to biofilm formation and virulence [31]. The genes of the denitrification pathway encoding the nitrite reductase (*aniA*, 3.3-fold), nitric oxide reductase (*norB*, 3.1-fold) as well as the nitrate ABC transporter complex (*nrtABCD*) were strongly upregulated after exposure to a 26°C cold shock. Interestingly, under conditions of cold shock the transcription of the repressor gene, *nsrR*, is also increased (4.8-fold), a mechanism that may prevent the uncontrolled overexpression of the denitrfication pathway genes. The upregulation of the denitrification genes after exposure of *M. catarrhalis* to 26°C may indicate that there is limited oxygen availability in this environment. Glutamine synthetase (*glnA*) involved in nitrogen assimilation was also up-regulated upon shift to cold shock conditions. In contrast, the genes encoding the two-component system nitrate/nitrite response regulator NarL and nitrate/nitrite transporters (*narK1/K2*) were down-regulated after exposure to 26°C. Similarly, many genes of *Y. pestis* involved in nitrogen assimilation were strongly upregulated upon shift to 26°C [32]. The nasopharyngeal mucosa is rich in nitric oxide (NO)-producing cells, including macrophages and epithelial cells, and local concentrations of NO have been shown to reach concentrations which are likely to be greatly in excess of that required to produce toxic effects in most bacteria [49]. The inreased ability to reduce nitric oxide after exposure to 26°C may also provide *M. catarrhalis* with some level of protection against macrophage-generated nitric oxide. The ability of *N. meningitidis* to survive in nasopharyngeal mucosal tissue has been shown to be enhanced by expression of nitric oxide detoxification systems [50].

### Differentially Expressed Genes Involved in Iron Acquisition

Our previous findings established that a 26°C cold shock upregulates the expression of genes involved in transferrin and lactoferrin acquisition, and enhances binding of these proteins on the surface of *M. catarrhalis* [13]. Analysis of RNA-seq data demonstrated that expression levels of genes involved in iron transport and utilization were significantly increased at 26°C in comparison to 37°C. The transcription of genes encoding transferrin binding proteins (*tbpA/B*, 1.7- and 2.4-fold respectively) and lactoferrin binding proteins (*lbpA/B*, 4.4- and 2-fold, respectively) was upregulated after exposure to 26°C. The *afeBCD* gene cluster, involved in the acquisition of chelated iron, was also upregulated after exposure to 26°C. In contrast, two bacterioferritins A/B (*bfrA/B*), involved in intracellular iron storage, were strongly (5.5- and 6.2-fold, respectively) down-regulated at 26°C. Efficient iron acquisition from lactoferrin is an important virulence factor for pathogenic mucosal bacteria. It has been shown that supplemental lactoferrin can enhance the virulence of meningococcal infection in mice [51]. Increased expression of *M. catarrhalis* lactoferrin and transferrin binding proteins following cold shock would facilitate the binding and acquisition of iron from lactoferrin/transferrin to support growth of bacteria in the mucosal environment.

### Differentially Expressed Genes Involved in Phosphate Metabolism

Expression levels of genes involved in phosphate transport and utilization were significantly increased at 26°C in comparison to 37°C. Examples are phosphate ABC transporter proteins (*pstABCS*) involved in the capture of periplasmic inorganic phosphate and its transport into the cytosol, and the PhoBR (*phoB/R*), a two-component regulatory system that appears to respond to external phosphate concentrations through interaction with a phosphate transport system and controls gene transcription of the Pho regulon [52]. Noticeably, phosphate ABC transporter periplasmic phosphate-binding protein PstS and phosphate transport system permease protein PstC stand out as the top two most upregulated genes in *M.catarrhalis* exposed to a 26°C cold shock, respectively. PstS was upregulated 110-fold, while PstC was upregulated 63-fold. Recent studies have shown that the Pho regulon plays a role in the virulence of several bacteria. The Pst system is found to be involved in modifications of lipid A and fatty acid composition [53]. An inactivating *pst* mutation in *E. coli* affects multiple virulence attributes; it reduces resistance to the bactericidal effect of serum, to acidity and to cationic antimicrobial peptides [54].

### Differentially Expressed Genes Involved in Sulfur Metabolism

Transcription of genes from the sulfur assimilation pathway (*cys DHNI*) were upregulated after exposure of *M. catarrhalis* to cold shock. Sulfur metabolic pathways are essential for survival and the expression of virulence in many pathogenic bacteria, including *Mycobacterium tuberculosis* [55]. The genes of the sulfate assimilation pathway are consistently upregulated in *M. tuberculosis* by diverse environmental cues, including nutrient starvation, hypoxia, oxidative stress, and cell wall stress [56].

### Differentially Expressed Genes Involved in Oxidative Stress

The superoxide dismutase-catalase system is able to counteract the effect of oxidative stress by catalyzing the conversion of superoxide to water and oxygen [57]. The expression of genes involved in oxidative stress response including superoxide dismutase A (*sodA*, 1.9-fold), catalase (*katA*, 2-fold), alkyl hydroperoxide reductase (*ahpC/F*, 4.3-and 3.6-fold, respectively), peroxiredoxin family protein/glutaredoxin (2.9-fold) was increased at 37°C. Activation of these genes suggests that *M. catarrhalis* may be exposed to reactive oxygen species (ROS) under 37°C conditions.

### Differentially Expressed Genes Involved in Nucleotide Metabolism

Nucleotides are important substrates for DNA synthesis and DNA repair. Analysis of RNA-seq data demonstrated that exposure of *M.catarrhalis* to 37°C caused significant induction of numerous genes involved in nucleotide metabolism (Figure 2B and Table S5). The majority of genes involved in purine (*purCHMN, guaB, prsA*) and pyrimidine (*pyrBDE, carA*) biosynthesis as well as genes involved in nucleotide and nucleoside interconversions (*gmk, pyrH, ndk*) were upregulated at 37°C. It has been shown that *de novo* nucleotide biosynthesis is critical for survival and growth of E. coli in human serum [58].

Many pathogenic bacteria respond to an increase from environmental temperatures to that of a mammalian body by the expression of genes that promote survival in the mammalian host [59]. Typically, these pathogens occupy at some stage of their life cycle an environmental reservoir such as soil, water, or an arthropod host from which mammalian hosts become infected. Bacterial species known to upregulate virulence gene expression in response to a shift to mammalian body temperature include *Yersinia*, *Borrelia*, *Shigella*, *Bordetella*, *E. coli* and *Streptococcus* [59]. Following a temperature upshift to 37°C, *B. pertussis*, *Y. enterocolitica* and *Y. pseudotuberculosis* activate the transcription of early genes that encode the factors required for bacterial adhesion [60], [61]. Similarly, the human body temperature increases in *E. coli* the expression of genes involved in adhesion as well as in iron and amino acids uptake and utilization [44].

*M. catarrhalis*, however, does not belong to the environmental microflora, but is an exclusively human mucosal pathogen and commensal. The *M. catarrhalis* cumulative nasopharyngeal colonization rate in young children is high (up to 75%) [1], [62], suggesting that the major source of transmission of *M. catarrhalis* to infants are adults and other young children. In contrast to other bacteria, a temperature downshift to 26°C activates the expression of the *uspA1* gene, a major adhesin of *M. catarrhalis*, and increases the adherence of *M.catarrhalis* to pharyngeal epithelial cells *in vitro* [12], [14]. Therefore, the relevance to pathogenesis of a highly adherent phenotype when bacteria are exposed to a 26°C cold shock merits discussion. In our previous studies we also found that a 26°C cold shock increases the proinflammatory effect on epithelial cells by enhanced release of the proinflammatory cytokine IL-8 [12]. Cold shock also affects the expression of genes involved in immune evasion: exposure to 26°C decreases the expression of hemagglutinin (Hag), a major adhesin, which mediates B cell responses, and reduces immunoglobulin D-binding on the surface of *M. catarrhalis*. This may reduce the stimulation of B cells and increase bacterial survival by prevention of endocytosis by these cells [13].

Cold shock-induced changes, which occur in the transcription of several membrane-related genes, demonstrate that *M. catarrhalis* may remodel its membrane components in response to a downshift of temperature. Many of these genes are known as major virulence factors. *M. catarrhalis* acquires many of the nutrients it requires for growth directly from the host. Under conditions of low temperature in the nasopharynx various nutrients may become limited as the metabolism of host cells slows. *M. catarrhalis* appears to respond by up-regulating proteins with homology to transporters and binding proteins, which are involved in the transport of nutrients from the host (iron, phosphate, amino acids).

We also found that a 26°C cold shock increases the expression of genes involved in the biosynthesis of pili leading to increased natural transformation rates. Competence for natural genetic transformation is a programmed physiologic state, which enables bacteria to take up and process exogenous DNA [63]. A rapid increase in the prevalence of β-lactamase-producing *M. catarrhalis* strains has been observed during in the last 30 years such that more than 95% of clinical isolates now appear to be resistant to one or more β-lactams [64]. Increased transcription of genes encoded the TFP and greater natural genetic transformation rates, which were observed after exposure of *M. catarrhalis* to a 26°C cold shock, may facilitate the rapid spread and acquisition of novel virulence-associated genes in *M. catarrhalis*, as has been shown for the *bro* β-lactamase gene, whose sequence suggested that it was acquired by interspecies gene transfer, possibly from a gram-positive bacterium [65], [66]. Recent studies reported an increase in resistance to trimethoprim/sulfamethoxazole (cotrimoxazole) of 18.5% in Taiwan and to 82.5% in India [67], [68]. Similarly, 80% of all *M. catarrhalis* strains tested in the UK and Ireland were resistant to cefaclor, and 5% to cefuroxime [69]. Furthermore, increased natural transformation following cold shock may also enhance the exchange of virulence properties within the carriage population, leading to new emergent phenotypes with heightened pathogenic potential. A recent study has shown that *M. catarrhalis* can incorporate the UspA1 CEACAM1-binding region not only into rare UspA1 proteins devoid of a CEACAM-binding region, but also into UspA2, which normally lack this capacity [70]. Conceivably, this could convey novel adhesive functions whilst enhancing immune evasion.

It is conceivable that cold shock could promote the virulence of *M. catarrhalis* by taking advantage of the induction of adherence and several uptake systems that in other bacteria have been related to virulence. Since it has been shown that one stress response might help bacteria to contend with other stress conditions [71], [72], it is possible that cold shock could improve the ability of *M.* *catarrhalis* to survive in the host because several pathways of stress responses are activated. Thus, cold shock induces in *M.* *catarrhalis* a complex of adaptive mechanisms that could convey novel pathogenic functions and may contribute to enhanced growth and colonization. Clinical studies in children have demonstrated that the density of *M. catarrhalis* in the nasopharynx is positively associated with prolonged respiratory tract symptoms and a greater likelihood of purulent otitis media [73], [74]. Therefore, the physiologic cold shock response may emerge as an important contributor to the seasonal peak in *M. catarrhalis* infections in temperate climates. Overall, these studies contribute a wealth of new information on the pathogenetic effect of a 26°C cold shock in *M. catarrhalis*.

## Supporting Information

Figure S1 **Reproducibility of the RNA-seq replicates.** Expression data for each biological replicate (26°C vs 37°C, n = 3) were plotted against each other.
(TIF)

Figure S2 **Differentially expressed genes following cold shock.** Correlation of fold-change and normalized mean expression (log scale). Genes showing significant differential expression in the RNA-seq data are highlighted in red, genes that are not regulated by temperature are highlighted in black.
(TIF)

Table S1 **Oligonucleotides used in this study.**
(DOC)

Table S2 **Summary of Illumina RNA-seq data.**
(DOC)

Table S3 **Differentially expressed genes according to DESeq analysis.** Genes overexpressed at 26°C in comparison with 37°C are at the top of this list, genes overexpressed at 37°C at the bottom. Genes are sorted according to their p-value.
(XLS)

Table S4 **Genes differentially expressed in response to cold shock.** Using a FDR cut-off of 5% a total of 831 genes were significantly differentially expressed greater than 1.5-fold, including 429 up-regulated genes and 402 down-regulated genes at 26°C.
(XLS)

Table S5 **Functional categories of** ***M.catarrhalis*** **genes induced or repressed following cold shock.** Frequency lists the number of genes present both in the gene set and in the specific category followed by the total number of reference genes in the category. Enrichment was tested using Fishers’s exact test (p-value) and adjusted for multiple testing using FDR (p-value adjusted). Categories with adjusted p-values<5% are highlighted in bold.
(DOC)
