# Correction: RNA-Seq-Based Analysis of the Physiologic Cold Shock-Induced Changes in *Moraxella catarrhalis* Gene Expression

**DOI:** 10.1371/annotation/a6e15b35-386c-4b0a-9de9-6fea091e9088

**Published:** 2013-12-20

**Authors:** Violeta Spaniol, Stefan Wyder, Christoph Aebi

A link forming the last sentence of the "RNA-seq Data Analysis" section of the Materials and Methods was incorrectly inserted. The correct last sentence is: "Enrichment analysis was performed using the functional categories with a custom python skript (Fisher’s exact test) correcting for multiple testing using the FDR."

In addition, a link was incorrectly inserted into the last sentence of the first paragraph of the "Differentially Expressed Genes Involved in Transcription and Replication" section of the Results and Discussion. The correct sentence is: "The ATP-dependent RNA helicase-encoding genes (rhlB), whose transcription was enhanced (1.9-fold) after temperature downshift, are known to be involved in the resolving of the cold-induced structuring of mRNA [27]."

