# Supplementary figures and images for: RNA-Seq-Based Analysis of the Physiologic Cold Shock-Induced Changes in Moraxella catarrhalis Gene Expression
Source: PLoS One. 2013 Jul 2;8(7):e68298. doi: 10.1371/journal.pone.0068298 (PMC3699543; doi:10.1371/journal.pone.0068298)

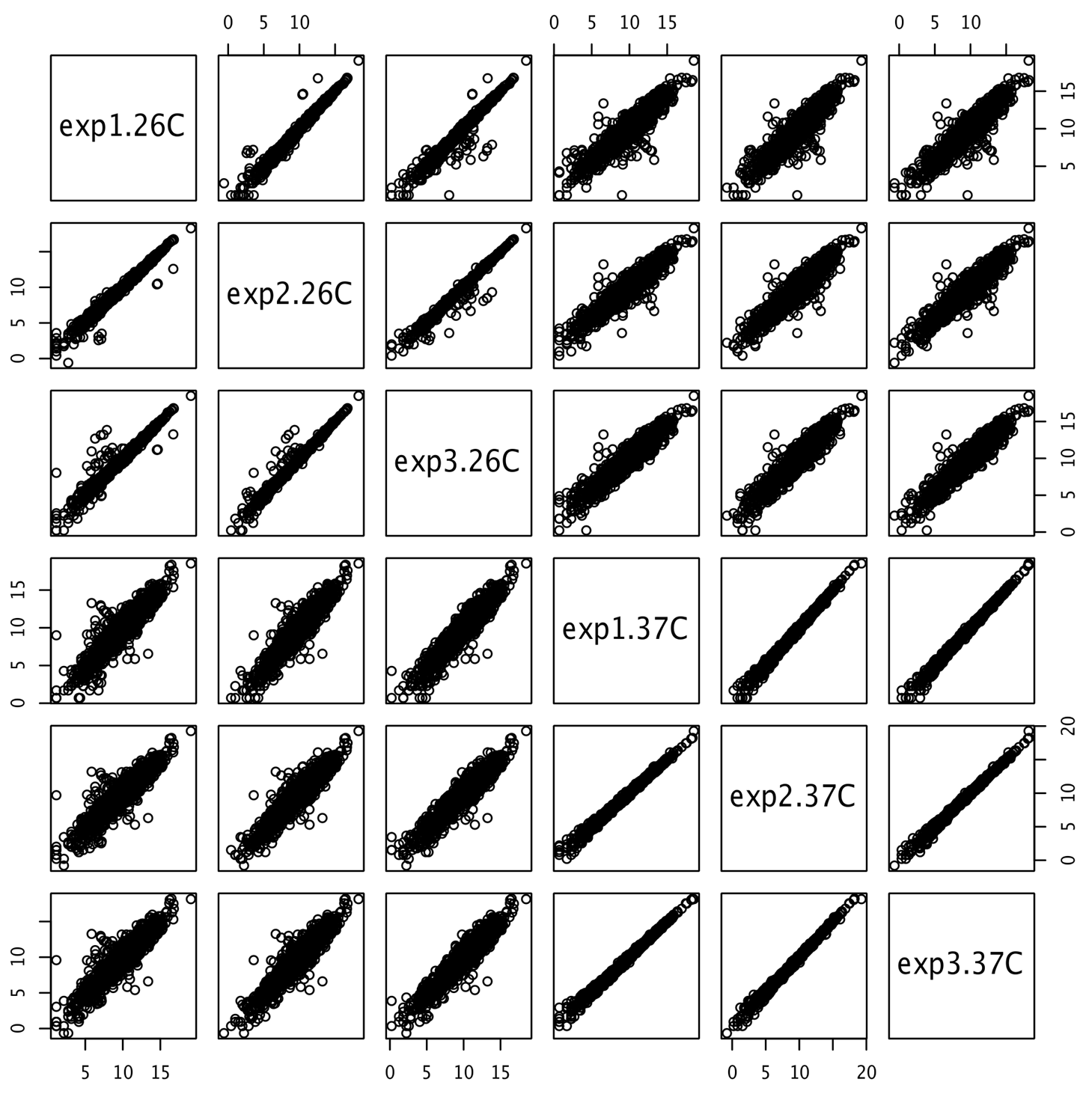

Supplement: Figure S1 — Reproducibility of the RNA-seq replicates. Expression data for each biological replicate (26°C vs 37°C, n = 3) were plotted against each other. (TIF) [file pone.0068298.s001.tif]

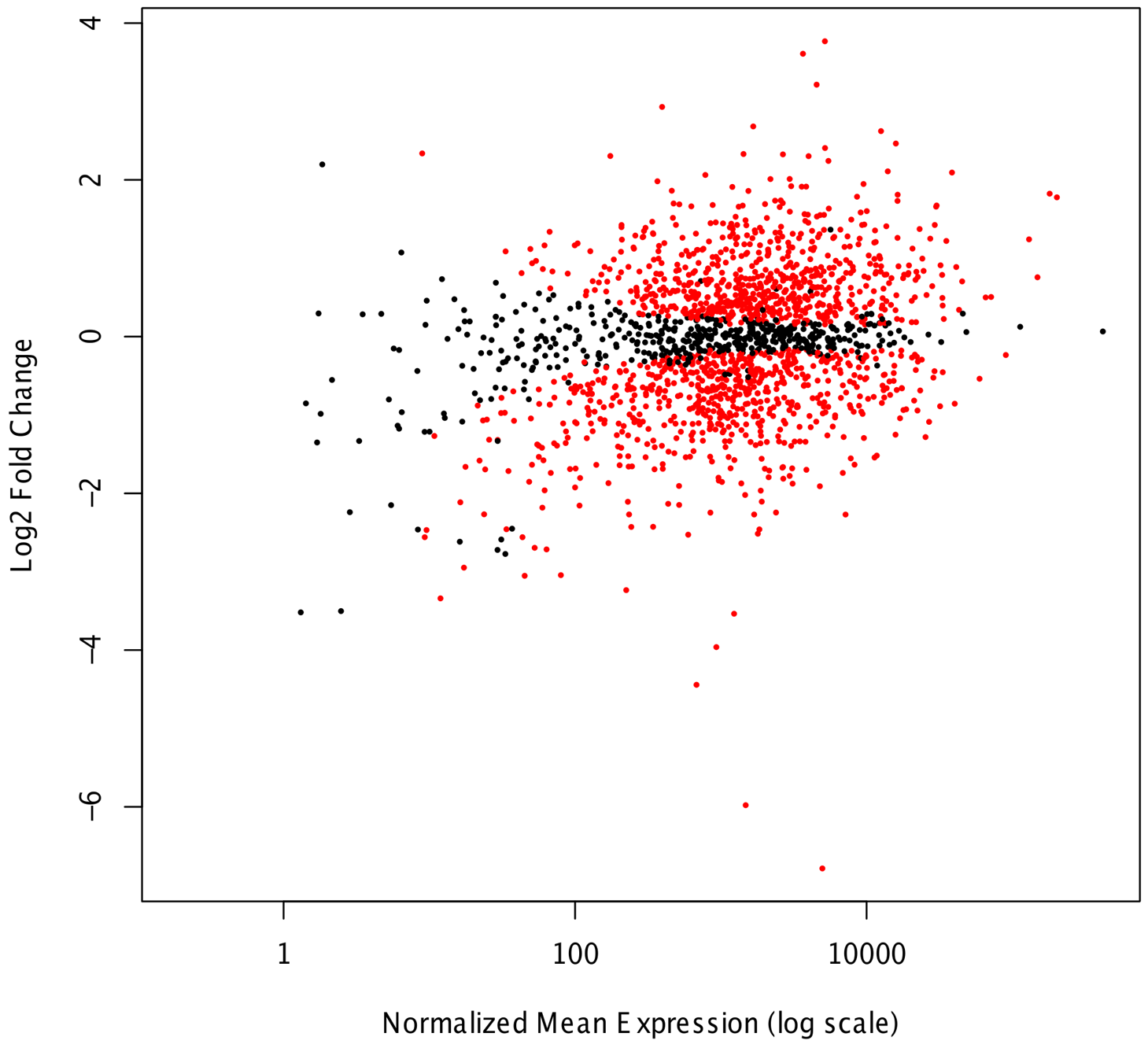

Supplement: Figure S2 — Differentially expressed genes following cold shock. Correlation of fold-change and normalized mean expression (log scale). Genes showing significant differential expression in the RNA-seq data are highlighted in red, genes that are not regulated by temperature are highlighted in black. (TIF) [file pone.0068298.s002.tif]
