# Supplementary material for: RNA-Seq-Based Analysis of the Physiologic Cold Shock-Induced Changes in Moraxella catarrhalis Gene Expression
Source: PLoS One. 2013 Jul 2;8(7):e68298. doi: 10.1371/journal.pone.0068298 (PMC3699543; doi:10.1371/journal.pone.0068298)
Supplement: Table S1 — Oligonucleotides used in this study. (DOC) [file pone.0068298.s003.doc]

Table S1. Oligonucleotides used in this study.

| **Name** | **Sequence (5’ – 3’)** |
| --- | --- |
| PstS_F | TATTCCGTTCAGACGGTTCA |
| PstS_R | CATTTGATGGCTTTGTCCAC |
| PstC_F | TGGTATGTGGGGTTTGTTTG |
| PstC_R | GCACCAACAAACATCTGACC |
| PhoR_F | ATCCGTCACCGACAGATGTA |
| PhoR_R | GGGCATTGAGGCTTATTTGT |
| PhoB_F | GGTAACTTTGGGTGGTCGTT |
| PhoB_R | CTTCCTCAATGACAGGCTCA |
| PilA_F | CGATGTTTGCCATACCTCAA |
| PilA_R | GCTGTCTGTAATGCCTTGGA |
| PliQ_F | GGCGACACCAAATATCACAC |
| PliQ_R | GATGACATTCGTTTCGATGG |
| NorB_F | GGTGAAGGCGATAAGAACCT |
| NorB_R | GCCAGTATTCCATGATGGCA |
| AniA_F | GCCAAAAAGCAGCACGGTA |
| AniA_R | GTTCTTGTGAGGCAGCATTATCTG |
| OMPA_F | TCAATACGACGGTTTTGGAA |
| OMPA_R | ATCAAAAGGTGTTGCTGCTG |
| M35-like porin_F | ATCTCTTGGCATTGCTGCTA |
| M35-like porin_R | TCAGACCCTCTAAGGCCAAT |
| OmpJ_F | TACCCCATTTTATGCCAAGG |
| OmpJ_R | CCTGCTAGGCTGGTTTTGTC |
| ompG1b_F | ATTTGGTGAAGTTGGTGCTG |
| ompG1b_R | GATGTCACGCTCTGCTTGTT |
| bfrA _F | CCCATCAAATCAATCAAACG |
| bfrA_R | AGAGGGTATCGCATTGTGTG |
| bfrB_F | AGTTCTTGTTGCGTGGTCAG |
| bfrB_R | GCGATTGGTATTTGTGAGGA |
| 16S RNA _F | CAATGGGCGAAAGCCTGAT |
| 16S RNA _R | GTGCTTTACAACCAAAAGGCCT |
