# Supplementary material for: RNA-Seq-Based Analysis of the Physiologic Cold Shock-Induced Changes in Moraxella catarrhalis Gene Expression
Source: PLoS One. 2013 Jul 2;8(7):e68298. doi: 10.1371/journal.pone.0068298 (PMC3699543; doi:10.1371/journal.pone.0068298)
Supplement: Table S2 — Summary of Illumina RNA-seq data. (DOC) [file pone.0068298.s004.doc]

**Table S2. Summary of Illumina RNA-seq data.**

| **Sample** | **Number of aligned reads** | **Number of non-rRNA reads** | **mRNA reads (% of all mapped reads)** |
| --- | --- | --- | --- |
| 26°C_1 | 13'807'058 | 3'214'746 | 23% |
| 26°C_2 | 27'880'294 | 9'966'862 | 36% |
| 26°C_3 | 17'127'887 | 5'791'186 | 34% |
| 37°C_1 | 19'038'969 | 5'038'161 | 26% |
| 37°C_2 | 66'832'479 | 14'576'839 | 22% |
| 37°C_3 | 38'898'117 | 12'527'014 | 32% |
