# Supplementary material for: RNA-Seq-Based Analysis of the Physiologic Cold Shock-Induced Changes in Moraxella catarrhalis Gene Expression
Source: PLoS One. 2013 Jul 2;8(7):e68298. doi: 10.1371/journal.pone.0068298 (PMC3699543; doi:10.1371/journal.pone.0068298)
Supplement: Table S5 — Functional categories of M.catarrhalis genes induced or repressed following cold shock. Frequency lists the number of genes present both in the gene set and in the specific category followed by the total number of reference genes in the category. Enrichment was tested using Fishers’s exact test (p-value) and adjusted for multiple testing using FDR (p-value adjusted). Categories with adjusted p-values<5% are highlighted in bold. (DOC) [file pone.0068298.s007.doc]

**Table S5. Functional categories of *M.catarrhalis* genes induced or repressed following cold shock.** Frequency lists the number of genes present both in the gene set and in the specific category followed by the total number of reference genes in the category. Enrichment was tested using Fishers’s exact test (p-value) and adjusted for multiple testing using FDR (p-value adjusted). Categories with adjusted p-values < 5% are highlighted in bold.

| **Functional category/**  **Specific function** | **Frequency** | **p-value** | **p-value adjusted** |
| --- | --- | --- | --- |
| **Upregulated at 26°C** |  |  |  |
| **Transcription**  Transcription factors | 16/35 (45.71%)  5/10 (50.00%) | 2.22E-03  5.55E-02 | **3.99E-02**  5.30E-01 |
| **Transport and binding proteins**  Anions | 46/146 (31.51%)  8/20 (40.00%) | 8.05E-03  6.50E-02 | **7.25E-02**  5.30E-01 |
| DNA metabolism  Restriction/modification | 23/77 (29.87%)  7/11 (63.64%) | 9.15E-02  4.37E-03 | 5.49E-01  1.31E-01 |
| Mobile and extrachromosomal element functions | 5/13 (38.46%) | 1.56E-01 | 7.01E-01 |
| Biosynthesis of cofactors, prosthetic groups, and carriers  Pantothenate and coenzyme A  Biotin | 24/90 (26.67%)  5/6 (83.33%)  4/5 (80.00%) | 2.26E-01  3.01E-03  1.11E-02 | 7.64E-01  1.31E-01  2.23E-01 |
| Regulatory functions | 9/31 (29.03%) | 2.65E-01 | 7.64E-01 |
| Amino acid biosynthesis  Glutamate family | 20/77 (25.97%)  6/14 (42.86%) | 2.97E-01  7.76E-02 | 7.64E-01  5.30E-01 |
| Cell envelope  Surface structures | 37/156 (23.72%)  6/12 (50.00%) | 4.33E-01  3.62E-02 | 9.75E-01  4.34E-01 |
| Central intermediary metabolism  Sulfur metabolism | 8/33 (24.24%)  4/6 (66.67%) | 4.95E-01  2.74E-02 | 9.90E-01  4.11E-01 |
| DNA transformation | 3/5 (60.00%) | 8.24E-02 | 5.30E-01 |
| **Downregulated at 26°C** |  |  |  |
| **Protein fate**  **Protein folding and stabilization** | 32/88 (36.36%)  13/23 (56.52%) | 7.38E-04  2.34E-04 | **1.33E-02**  **1.41E-02** |
| **Purines, pyrimidines, nucleosides, and nucleotides**  Purine ribonucleotide biosynthesis  Pyrimidine ribonucleotide biosynthesis | 16/36 (44.44%)  6/14 (42.86%)  4/8 (50.00%) | 1.54E-03  5.89E-02  7.03E-02 | **1.38E-02**  5.05E-01  5.27E-01 |
| Energy metabolism  **Glycolysis/gluconeogenesis**  Electron transport | 38/140 (27.14%)  9/16 (56.25%)  19/59 (32.20%) | 5.80E-02  2.38E-03  3.41E-02 | 3.48E-01  **4.77E-02**  3.41E-01 |
| Cellular processes  Detoxification | 17/60 (28.33%)  6/10 (60.00%) | 1.25E-01  8.98E-03 | 4.67E-01  1.08E-01 |
| Fatty acid and phospholipid metabolism  **Biosynthesis** | 12/40 (30.00%)  10/21 (47.62%) | 1.30E-01  6.62E-03 | 4.67E-01  **9.94E-02** |
| Signal transduction | 4/11 (36.36%) | 1.94E-01 | 5.83E-01 |
| Central intermediary metabolism | 9/33 (27.27%) | 2.64E-01 | 6.00E-01 |
| Unknown function | 39/165 (23.64%) | 2.67E-01 | 6.00E-01 |
| Biosynthesis of cofactors, prosthetic groups, and carriers  **Molybdopterin** | 21/90 (23.33%)  6/7 (85.71%) | 3.70E-01  5.44E-04 | 7.40E-01  **1.63E-02** |
